# Effects of Using Corn Dried Distillers’ Grains with Solubles (cDDGS) as a Partial Replacement for Soybean Meal on the Outcomes of Pig Fattening, Pork Slaughter Value and Quality

**DOI:** 10.3390/ani11102956

**Published:** 2021-10-14

**Authors:** Tomasz Schwarz, Marcin Przybyło, Piotr Zapletal, Artur Turek, Mariola Pabiańczyk, Pawel Mieczyslaw Bartlewski

**Affiliations:** 1 Department of Animal Genetics, Breeding and Ethology, University of Agriculture in Kraków, 24/28 Mickiewicza Ave., 30-059 Cracow, Poland; piotr.zapletal@urk.edu.pl (P.Z.); mariolka_pabianczyk@wp.pl (M.P.); 2 Department of Animal Nutrition and Biotechnology and Fisheries, University of Agriculture in Kraków, 24/28 Mickiewicza Ave., 30-059 Cracow, Poland; przybylo.mk@gmail.com; 3 Blattin Poland, Siedlec, Poznowicka St. 1, 47-180 Izbicko, Poland; aturek.blattin@gmail.com; 4 Department of Biomedical Sciences, Ontario Veterinary College, University of Guelph, 50 Stone Rd., Guelph, ON N1G 2W1, Canada; pmbart@uoguelph.ca

**Keywords:** soybean meal, grower, finisher, weight gain, FCR, profitability

## Abstract

**Simple Summary:**

Soybean meal is the principal component of the diets for fattener pigs; however, there is a constant need for alternative, inexpensive, preferably non-GMO (genetic modified organisms) protein sources in swine nutrition. Corn dried distillers’ grains with solubles (cDDGS), by-products of ethanol and bioethanol production, are one such alternative. Due to their chemical composition, they cannot completely replace soybean meal; however, even relatively small additions of cDDGS may decrease the cost of feeding and increase profitability of pig production. In this study, the pigs were fed two different diets: control (cereal–soybean meal-based) or cDDGS (with soybean meal partially replaced with cDGGS). The growth performance as well as carcass and meat quality traits of fatteners fed cDGGS-containing diets did not differ from those of their respective controls. However, the addition of even 15% of cDDGS did result in lower production costs and increased the profitability of pig fattening.

**Abstract:**

The present study set out to determine the effects of incorporating cDDGS into starter, grower, and finisher diets (containing 5%, 10%, and 15% of cDDGS, respectively) on growth performance, carcass and meat quality, and cost effectiveness of pig fattening. Sixty-four pigs (mean body weight of 15.0 ± 2.1 kg) were divided into two groups (*n* = 32) and fed a control diet (cereal–soybean meal-based) or cDGGS-containing diets (with soybean meal partially replaced with cDDGS). Live weights of pigs as well as weight gains/daily weight gains across all fattening phases did not differ between the two groups of fattener pigs studied (*p* > 0.05). Addition of cDDGS decreased feed intake per pig during the grower (*p* < 0.05) and finisher (*p* < 0.01) phases, and, as a result, throughout the entire fattening period (254 vs. 245 kg for control and cDDGS groups, respectively; *p* < 0.01). The feed conversion ratio (FCR) for the entire fattening period was significantly less for cDDGS-fed fatteners (2.77) than for controls (2.91; *p* < 0.05). Carcass weights, fat thickness, and meatiness did not vary between the two groups of animals (*p* > 0.05). Loin depth was greater in the cDDGS group by ~5 mm (*p* < 0.05). Slaughter value was higher for the cDDGS group (76.1% vs. 77.0%, *p* < 0.05). The total cost of fattening and total cost of 1 kg of body weight decreased in cDDGS compared with the control subset of fatteners by ~7% and 8% during the grower and finisher phases, respectively (*p* < 0.01). The simplified direct surplus per pig was approximately 63% higher for the cDDGS group. Our results indicate that even moderate inclusion of cDDGS to concentrate mixtures (or a partial replacement of soybean meal with cDDGS) may improve FCR without any substantial changes in meat and back fat characteristics as well as significantly decrease the cost of feeding and increase the profitability of pig production.

## 1. Introduction

Soybean meal (SBM) is the principal component of the diets for fattener pigs due mainly to its high protein content and quality, lysine-rich amino acid profile, and relatively low levels of anti-nutrients [1]. However, there is a constant need for alternative, low-cost protein sources in swine nutrition, preferably non-GMO and/or sustainable feed components. Dried distillers’ grains with solubles (DDGS), by-products of ethanol and bioethanol production, are one such alternative. Corn is commonly processed in distilleries and so corn DDGS (cDDGS) are widely available for use in livestock nutrition. Concentrations of protein, fiber, and fat are approximately 3 times higher in cDDGS compared with corn grain [2]. Corn dried distillers’ grains with solubles are also a good source of digestible phosphorus and energy [2,3]. As a source of dietary protein, cDDGS are less expensive than soybean meal, but they contain less crude protein and essential amino acids, especially digestible lysine [3]. A high level of low-digestibility fiber (~42%) results in reduced digestibility or utilization of dry matter (DM) and energy from cDDGS [3]. Although high levels of oil in cDDGS increase their energy value, the abundance of unsaturated fatty acids, particularly linoleic acid, may result in “softening of the fat” when fed to the finisher pigs [3,4].

High dietary value and positive consumers’ perception (e.g., sensory attractiveness) are key “ingredients” of meat quality and acceptance, both raw and cooked [5]. Fresh pork should be greyish-pink in color (as meat sits in the refrigerator or freezer, its color can become slightly lighter or darker) and have a “shine” that is not associated with an excessive leakage of juice. Color is one of the most important criteria in consumers’ assessment of meat quality and freshness [6,7]. Inappropriate color of meat and meat products, usually associated with pungent smell and slimy texture, effectively discourages a potential buyer. The color should be stable and should not turn to gray when the meat is in contact with atmospheric air. The meat should have a specific smell and slightly sour taste as well as a firm texture. A low level of marbling is optimal, as it ensures desirable taste and aroma after the thermal treatment. The influence of pig fattening with cDDGS on meat quality, and especially its sensory characteristics, has not been elucidated. Previous studies using up to 20–30% of cDDGS in the diets for growing–finishing pigs have mainly focused on carcass meatiness as well as fatty acid profiles and color of the meat [3,4,8,9,10,11]. Most recent studies have shown that inclusion of even 45% of cDDGS in feed for growing–finishing pigs did not adversely affect their growth performance or carcass meatiness, but it did increase polyunsaturated fatty acid content and the softness of fat [4]. However, due to its relatively low cost, cDDGS are still an attractive alternative to corn grain or soybean meal in swine nutrition, being especially popular in the USA [3] but not in central Europe, with less optimal environmental conditions for corn cultivation and fewer distilleries producing cDDGS. The cost of funding and operating such distilleries remains rather high. Differences in climatic influences and production procedures result in substantial differences in quality parameters of corn and cDDGS between central Europe and North America [4]. Consequently, the results of research on DDGS conducted in the USA are not directly applicable to the situation in the European agri-food sector.

The general aim of this study was to determine the effects of starter, grower, and finisher diets containing 5%, 10%, and 15% of cDDGS of central European origin on growth performance, carcass and meat quality, and cost effectiveness of pig fattening. We hypothesized that cDDGS as a partial replacement for soybean meal for growing–finishing pigs would not have negative effects on meat and carcass quality traits but that the addition of even low/moderate amounts of cDDGS would decrease feed cost and increase the profitability of pig fattening.

## 2. Materials and Methods

The present study complied with the EU Directive 2010/63/EU for animal experimentation and the Polish law for the care and use of experimental animals (2 August 1997), and the study was approved by the first local Animal Care and Ethics in Research Committee at the Jagiellonian University, Cracow, Poland (decision no. 90/2012).

### 2.1. Animal Nutrition and General Experimental Considerations

All grains (wheat, barley, and triticale) as well as soybean meal and corn dried distilled grains with solubles (cDDGS) were initially examined by the Blattin Poland analytical laboratory (Siedlec, Poland). The samples were analyzed by Fourier transform near-infrared (FT-NIR) spectroscopy, as described earlier [12,13]. Each sample was exposed to electromagnetic radiation in the near-infrared range that is absorbed and causes the vibrations of samples’ chemical bonds. These vibrations alter the output signal reaching the detector such that it generates readable information about the chemicals contained in the sample. The built-in software analyzes the points that are specific to different chemical bonds and identifies individual chemical compounds with high accuracy. Then, the sample spectra are compared with a mathematical model created during the calibration of the device to determine the qualitative and quantitative chemical composition of the sample. Based on the results of spectroscopic analyses (crude ash, protein, fat, fiber, and starch content; Table 1), the composition of all diets (complete mixtures) was calculated in compliance with DLG standards (DLG 2011). The diets were formulated to be isocaloric and isonitrogenous; only slight differences between the two diets (not exceeding 1–2%) were revealed after laboratory analyses (Table 2).

The control diet contained wheat, barley, triticale, SBM, soybean oil, and an additive (mineral–vitamin premix for fattening pigs; Blattivit PIC Ekonomast, Blattin Poland, Siedlec, Poland). It contained 13.6, 13.2, and 12.7 MJ ME and 177, 173, and 141 g of crude protein per 1 kg of feed mixture in the starter, grower, and finisher diets, respectively. In the experimental diets (cDDGS group), SBM meal was replaced by cDDGS in the amounts of 5, 10, and 15% of SBM in starter, grower, and finisher diets, respectively (Table 2). It contained 13.4, 12.9, and 12.6 MJ ME and 175, 171, and 140 g of crude protein per 1 kg of feed mixture in the starter, grower, and finisher diets, respectively. Proportions of ingredients were changed between the starter, grower, and finisher diets to adjust them to normative needs of animals and to attain optimal market price of the feed (Table 2).

The present study was carried out in a small commercial facility located in Komparzów, central Poland, and the study utilized 64 purebred fattener pigs of Polish Landrace genotype, purchased at 15.18 ± 2.05 kg of body weight and divided by sex, weight, and body condition core into two equal nutritional groups (*n* = 32; control: 15 females and 17 castrated males; and cDDGS group: 14 females and 18 castrated males). The animals were housed in the same litter-bedded building divided into 12 pens (5–6 pigs per pen), were fed *ad libitum* using dry Domino feeders (Domino Co., Torring, Denmark), and had unrestricted access to drinking water. The fattening period lasted 125 d and was divided into 3 phases—namely, starter, grower, and finisher phases, lasting 18, 45, and 62 d, respectively. Live weights of animals were taken at the outset of the experiment as well as after each fattening phase and before slaughter. The amount of feed given was recorded for every pen for the ensuing calculation of the feed conversion ratio (FCR).

After fattening, all animals were slaughtered in a local abattoir, and slaughter value was determined immediately after evisceration and weighing the carcasses. The lean meat content was assessed using an Ultra-Form 300 apparatus (Carometec Demark, Herlev, Denmark). Methodology for the assessment of lean meat content was outlined in the Commission Decision of 11 March 2005 (no. C(2005) 552), authorizing the methods for grading pig carcasses in Poland; https://eur-lex.europa.eu/legal-content/EN/TXT/PDF/?uri=CELEX:32005D0240&qid=1632488908630&from=PL, accessed on 7 October 2021). The carcasses were assigned to different classes based on the SEUROP standard system, comprising six classes varying in meat content by ~5% (from class S, over 60% of meat, to class P, <40% of meat). Subsequently, the analysis of production efficiency was completed based on the present fattening and slaughter data combined with market prices obtained from the Polish Ministry of Agriculture and Rural Development (https://www.gov.pl/web/rolnictwo/notowania-w-2021-r5; accessed on 20 April 2021). The price of different carcass classes was as follows: S class, PLN 5.61 (USD 1.52); E class, PLN 5.52 (USD 1.50); U class, PLN 5.21 (USD 1.41); R class, PLN 4.93 (USD 1.34); O class, PLN 4.40 (USD 1.19); and P class, PLN 3.65 (USD 0.99). All costs were initially calculated in Polish currency (PLN) but were then converted to USD to provide a clearer comparison to international readers [12,13].

### 2.2. Meat Quality Assessment

After 24 h of carcass storage at 4 °C, thirty samples of loin (*musculus longissimus dorsi*-LD) with back fat (excised at the level of the 1st–3rd lumbar vertebrae) were collected for laboratory analyses (15 randomly chosen samples per group). Main physicochemical and sensory characteristics were determined in raw and roasted meat. Meat acidity was measured with a pH meter equipped with a Metron OSH 12-00 electrode 24 h after slaughter in the right LD muscle at the level of the last thoracic vertebrae. Dry matter, protein, and fat content were determined with standard methods described in AOAC (2005). Free water content was determined by subtracting the mass of a muscle after 24 h of storage in a foil bag at 4 °C from its original mass (i.e., before storage) [14]. Thermal drip was determined by comparing muscle mass before and after roasting. The color components were measured using the CIE L* a* b* scale on the freshly cut surface of the muscle (after 20 min of conditioning at 4–6 °C, also known as blooming time) and after roasting, using a Konica Minolta CM2600d spectrophotometer (D65 light source, 10° observer, 8 mm measuring head aperture, calibrated on a white standard: L* 99.18, a* −0.07, b* −0.05). Each measurement was done in five replicates [15]. To determine cutting force and perform texture profile analysis (TPA) [16,17], muscle samples were cut along the muscle fibers to form cylinders with a diameter of 2.54 cm and a height of 2 cm. The samples were individually vacuum packed and then frozen in a freezer for 72 h at −18 °C. After thawing, one half of each sample was roasted at 180 °C to an attainment of internal temperature of 78 °C, and the other half was not heat treated. The cutting force was measured using a TA-XT2 apparatus from Stable Micro Systems, with a Warner-Bratzler attachment equipped with a triangular cutout knife. The speed of knife movement during the test was 1.5 mm/s, and the results are presented as the value of the cutting force (N) and as a function of sample shearing (N/s). Texture profile analysis (TPA) included the determination of hardness, springiness, resilience, adhesion, cohesion, and chewiness, and it was carried out using the same apparatus with a cylindrical attachment (diameter of 50 mm). The test of double compression of the samples to 70% deformation of their original height was performed. The roller speed was 2 mm/s, the pressure interval was 3 s, and the detection threshold was 10 g. Lastly, the sensory evaluation of meat samples after the heat treatment was carried out using a scale from 1 to 5 points (1 point = lowest rating) to determine their general external appearance, color, structure, smell, juiciness, tenderness, and taste [18]. A seven-person consumer panel blindly analyzed the sensory parameters of LD muscle samples.

### 2.3. Statistical Analyses

The production parameters of interest are listed in Table 3, Table 4 and Table 5, economic variables are summarized in Table 6, and meat quality parameters are listed in Table 7, Table 8 and Table 9. All data were initially subjected to heterogeneity and equal distribution test (Shapiro test). To assess the effect of cDDGS addition to the diet, the differences between cDDGS and control groups were analyzed by Student’s *t*-test for independent groups, except for the sensory parameters, which were analyzed by paired Student’s *t*-test. Data presented in Table 3 and Table 5 were analyzed on a per animal basis (*n* = 31 and *n* = 31 animals/group; one animal in each group died by the end of the fattening period). Proportions of pig carcasses in different classes were compared between the groups by χ^2^-test (Brandt and Snedecor formula for comparison of proportions; [19]). Data presented in Table 4 and the first part of Table 6 were calculated for all animals placed in a single pen (pen-level determination) since the values for feed intake and conversion rates in the group housing system could not be determined for individual animals. Calculations of direct surplus were performed to evaluate the profitability of production for both groups of fatteners (descriptive statistics; the second part of Table 6). Data in Table 7 and Table 8 were calculated for the meat samples taken (*n* = 15/group), whereas data in Table 9 were analyzed on the basis consumption panel reports (*n* = 7). All data were analyzed using Statistica ver. 12 software (Statsoft Poland, Cracow, Poland). All results are presented as means ± standard deviations (SDs) unless otherwise indicated.

Main sensory characteristics of the roasted loin analyzed by the consumer panel are summarized in Table 9. None of the sensory attributes differed between the two groups of fattener pigs studied (*p* > 0.05).

## 3. Results

Live weights of pigs as well as weight gains/daily weight gains in all fattening phases did not differ between the two groups of fattener pigs (*p* > 0.05; Table 3 and Table 4). Daily weight gains of pigs averaged 725 g, and animals were slaughtered at ~106 kg of body weight. Addition of cDDGS decreased feed intake per pig during the grower (*p* < 0.05) and finisher (*p* < 0.01) phases and during the entire fattening period (254 vs. 245 kg for control and cDDGS, respectively; *p* < 0.01). The FCR for the entire fattening period was less for cDDGS (2.77) than for control (2.91; *p* < 0.05).

Summary of the slaughter values, SEUROP classification, and prices of carcasses are given in Table 5. Live and carcass weights, fat thickness, and meatiness did not vary between the two groups of animals (*p* > 0.05). Slaughter value was higher for cDDGS group (76.1% vs. 77.0%, *p* < 0.05). Loin depth was greater for cDDGS group by ~5 mm (*p* < 0.05). According to the SEUROP classification system, more than 77% of carcasses from the cDDGS group were classified as S or E class as opposed to 58% of carcasses in the control group (*p* < 0.05), and none of them were classified as P class (3% in SBM). Consequently, the average carcass price per 1 kg was higher for cDDGS (PLN 5.43/USD 1.47) than for control (PLN 5.29/USD 1.43; *p* < 0.05).

The final cost of nutrition and profitability of pig fattening are summarized in Table 6. Inclusion of cDDGS into the grower and finisher diets decreased the feeding cost per pig by about 5% and 8%, respectively (*p* < 0.01). As a result, the total cost of fattening and total cost of 1 kg of body weight also decreased by about 7% and 8% during the grower and finisher phase, respectively (*p* < 0.01). The simplified direct surplus per pig was higher by ~63% for the cDDGS group.

Basic physicochemical characteristics of raw loin and the quality of backfat in the fattener pigs of the present study studied are summarized in Table 7. Mean pH of LD (5.65–5.67) measured 24 h after slaughter did not differ significantly between the two nutritional groups of pigs. The meat of the animals from the control group had a significantly higher value of the L* component (color brightness) compared with the cDDGS group. The muscles of the fatteners from the experimental group exceeded (*p* < 0.05) control muscles in free water content. The content of basic chemical components of the meat was significantly higher in the experimental group compared with controls. The meat of the fattener pigs in the experimental group also had significantly greater texture parameters—namely, hardness, elasticity, and chewiness.

Main physicochemical attributes of LD after roasting are given in Table 8. Significantly greater shear force and thermal drip as well as dry matter and protein content were observed in the experimental group of fattener pigs. However, control animals significantly exceeded cDDGS fatteners in meat cohesiveness, resilience, and extractable fat content. The meat of the control animals after roasting was also significantly more yellow (color channel b*) compared with the experimental group.

## 4. Discussion

The present results indicate that the addition of cDDGS (5%, 10%, and 15% in the starter, grower, and finisher phases of pig fattening, respectively) improved FCR without any substantial changes in meat and back fat characteristics. Although earlier studies have shown that much higher proportions of cDDGS may effectively be used in pig fattening [4], the present experiment has shown that even moderate amounts of cDDGS added to concentrate mixtures (or a partial replacement of soybean meal with cDDGS) may significantly decrease the cost of feeding and hence increase the profitability of production.

Soybean meal is the most commonly used protein component of feed mixtures for growing pigs [20]. The alternative sources of dietary protein, especially less expensive sources, are in great demand. However, cDDGS are not consistently regarded as the first-choice alternative to soybean meal, as their net protein content is half of that in soybean meal (Table 1). Despite this difference, and as shown in the present study (Table 2), cDDGS can still be incorporated into the feed mixtures for pigs, even those composed of only a few main ingredients, without any significant alterations in chemical composition of formulated mixtures. The differences in energy levels as well as protein and amino acid content between the control and experimental diets in this study were negligible. The level of amino acids in cDDGS (i.e., lysine, threonine, and tryptophan) should be monitored only if the proportion of cDDGS is greater than 10% [21]. Consequently, even farmers with only elementary knowledge of diet formulation can use cDDGS supplemented at relatively low proportions. Alternatively, inclusion of greater proportions of cDDGS may result in a pronounced deficit of nutrients, which in turn may necessitate the supplementation of essential amino acids, thus increasing the cost of feeding [4]. Considering the current market prices of soybean meal, cDDGS, and synthetic amino acids in Europe, the addition of up to 30% of cDDGS to the feed mixture (as a soybean replacer) is still profitable (data not shown; calculations completed on the day of paper submission).

In this study, inclusion of cDDGS resulted in a decreased cost of fattening (per fattener pig and per 1 kg of weight gain); this was due mainly to a reduced cost of the grower and, particularly, finisher phase of fattening. The use of cDDGS (and DDGS in general) during the starter phase is limited because of their relatively low protein quality and elevated fiber level as well as the higher nutritional requirements of younger fattener pigs (DLG 2011). However, feed intake in the starter phase is generally lower compared with the grower and finisher phases, and thus only the latter two phases impinge on the total cost of feeding. Moreover, the carcasses of pigs fed cDDGS-containing diets were of better quality according to the SEUROP scoring system, and their price per kg increased by nearly 3% (PLN 5.43 vs. PLN 5.29). Albeit not statistically significant, the use of cDDGS generated an almost USD 10 higher surplus per pig. This is important, especially considering the current global destabilization of the pork industry by African Swine Fever.

Daily weight gains did not vary between the two nutritional planes throughout the entire study. However, feed intake was lower in the grower and finisher phases in the cDDGS group. Consequently, FCR for the entire fattening period was lower for cDDGS fattener pigs compared with their control counterparts (2.71 vs. 2.91). Numerous studies have investigated the effect of DDGS in growing–finishing pigs on their average daily gains, feed intake, and FCR [21]. In most such studies, the average daily gains, feed intake, and FCR were not affected by cDDGS added to concentrate mixtures [22]. A lack of effect of cDDGS on daily gains, feed intake, and FCR was also observed when diets were based on cereals (e.g., corn) and soybean meal, and pigs received up to 15% of DDGS [8]. It was also shown that diets containing up to 20% [9] or even 30% [23] of cDDGS had no adverse effect on pig performance, provided that essential amino acids were added to the formulation. As in our present study, Xu et al. [24] observed a decreased feed intake but improved FCR in growing–finishing pigs fed corn–soybean meal diets containing up to 30% of cDDGS. Decreased feed intake in fattener pigs receiving cDDGS-containing diets was also noted in other studies; however, it was typically associated with reduced daily weight gains of animals [25]. It has been proven that pigs prefer corn–soybean meal diets over cDDGS diets, and increasing the level of cDDGS linearly decreased overall feed intake [26], suggesting that cDDGS is a less palatable component of diets for pigs.

Interestingly, inclusion of cDDGS into the diets for the fatteners of the present study increased the dressing percentage without affecting live or carcass weights. The difference was small but significant (77.0 vs. 76.1 in cDDGS and control, respectively). According to several studies summarized by Stein and Shurson [3], inclusion of cDDGS has no effect on or slightly decreases the dressing percentage, which contrasts with our present results. The decrease in dressing percentage may be explained, at least partly, by the high fiber level in DDGS compared with other components (cereals, soybean meal), which leads to increased gut fill in pigs fed DDGS-containing diets [27]. In our study, loin depth was another outcome improved by dietary cDDGS inclusion (~5 mm difference). DDGS in the diets for fatteners do not normally affect loin depth [3]. However, in most of the earlier studies, DDGS inclusion resulted in lower daily weight gains, and thus pigs were slaughtered at a lower body weight (mean increases in body muscle size and body weight are greatest during the final stages of the fattening). It can, therefore, be suggested that with a maximum content of cDDGS up to 15% of feed ration, they do not hamper weight gains. These proportions of cDDGS appear to be optimal in the dry feeding system for fattener pigs.

Pork meat should have a pH value in the range of 5.7–5.8 [28]. In the LD samples of the fattening pigs from the experimental and control groups, no PSE meat was found (pH 24 h ≤ 5.5), confirming the proper course of the glycolysis process in muscles. These results are in complete agreement with those in the available literature [28,29,30,31,32,33]. The meat of fatteners fed cDDGS-containing diets was characterized by greater natural and thermal loss, which worsened its textural parameters, such as hardness and firmness.

Nutrition is one of the extrinsic factors that can cause changes in the microstructure of the skeletal muscle, cytoskeleton of myocytes, and size of collagen fibers [34]. Mean age, body weight [35], and genetics [36,37,38] can also impinge on the histophysiological characteristics of muscle fibers. Ultimately, changes in the composition of muscle fibers not only can modulate the physiological parameters of the entire muscle but also can affect its histochemical profile (i.e., meat quality [39,40,41]). A criterion that plays a pivotal role in assessing the quality of meat is its color, because it dictates the preferences of consumers at the time of meat purchase [42]. In this study, mean values of the a* color component in the CIE Lab system were lower compared with those presented by Lisiak et al. [43] and Karpesiuk et al. [17], using intensively fed fatteners. The brightness of the raw LD muscle in the experimental group was less and the redness (a*) was greater than that in control group of fatteners, which according to consumers is a favorable feature [44]. In a study by Świątkiewicz et al. [11], the meat of pigs fed a mixture containing cDDGS had slightly lower values for lightness, redness, and yellowness, but the differences were not statistically significant from those obtained in the control group. Widmer et al. [9] observed a linear decrease in the meat yellowness of DDGS-fed pigs and no effect on its brightness. Wang et al. [45] observed no effect of cDDGS on meat color. In all, our present results and those obtained in other studies can be collectively interpreted to conclude that the use of cDDGS does not have any adverse effects on the color parameters of pork as determined in the CIELab model. Furthermore, in the general assessment of sensory characteristics, meat samples from both nutritional groups received a good score, and no significant differences were found between the two groups of fatteners.

## 5. Conclusions

Corn dried distillers’ grains with solubles, as a partial replacement (up to 15%) for soybean meal for growing–finishing pigs, had no effect on weight gain but decreased feed intake and improved FCR without any substantial changes in meat and back fat characteristics. Inclusion of even small/moderate amounts of cDDGS to concentrate mixtures for pigs (or a partial replacement of soybean meal with cDDGS) may significantly decrease the cost of feeding and hence increase the profitability of production.

## Figures and Tables

**Table 1:** Chemical composition of main feed ingredients.

Chemical Composition (g)	Feed Ingredient				
	Wheat	Triticale	Barley	cDDGS	Soybean Meal
Crude ash	17.2	16.5	20.7	52.6	58.2
Crude protein	138.2	111.3	122.1	278.4	462.8
Crude fat	15.1	12.9	18.9	94.4	21.1
Crude fiber	23.5	27.2	44.8	72.8	38.9
Starch	656.2	651.5	579.5	112.8	39.8

**Table 2:** Main ingredients, chemical composition, and metabolic energy of the experimental (SBM + cDDGS) and control diets.

Item	Starter		Grower		Finisher	
	cDDGS	Control	cDDGS	Control	cDDGS	Control
Ingredients (%)						
Soybean meal	18.00	21.00	13.00	18.00	4.00	10.00
DDGS	5.00	–	10.00	–	15.00	–
Wheat	42.00	42.00	35.00	32.00	–	–
Barley	30.00	32.00	30.00	36.00	48.00	57.00
Triticale	–	–	8.00	10.00	30.00	30.00
Soybean oil	1.00	1.00	1.00	1.00	–	–
Minerals/vitamins	4.00	4.00	3.00	3.00	3.00	3.00
Chemical composition						
Dry matter (%)	87.50	87.54	87.60	87.52	87.58	87.46
Crude ash (%)	6.60	6.60	5.58	5.38	5.27	4.59
Crude protein (%)	17.52	17.74	17.08	17.27	14.03	14.08
Crude fat (%)	2.87	2.71	3.27	2.54	2.34	1.62
Starch (%)	48.06	45.14	51.53	48.24	55.24	52.85
Monosaccharides (%)	3.80	3.92	3.36	3.73	2.78	3.10
Fiber (%)	3.78	3.42	3.57	3.19	4.09	3.71
Calcium (g/kg)	8.32	8.07	9.00	8.38	8.15	6.39
Phosphorus (g/kg)	5.95	5.14	5.69	5.34	5.48	4.88
Lysine (%)	1.09	1.12	0.98	1.08	0.83	0.86
Methionine + cysteine (%)	0.65	0.63	0.60	0.65	0.53	0.57
Threonine (%)	0.76	0.76	0.68	0.69	0.57	0.56
Tryptophan (%)	0.20	0.21	0.20	0.22	0.17	0.18
Metabolic energy (MJ/kg)	13.38	13.59	12.92	13.16	12.64	12.74

**Table 3:** Bodyweights at control weighing and weight gains of pigs during fattening (means ± SDs).

Variable	cDDGS (*n* = 31)	Control (*n* = 31)
Live weights (kg)		
Initial bodyweight	15.1 ± 2.1	15.3 ± 2.0
Bodyweight after starter phase	24.9 ± 3.1	24.7 ± 2.7
Bodyweight after grower phase	55.2 ± 5.5	54.6 ± 5.2
Bodyweight before slaughtering	106.4 ± 7.9	105.5 ± 12.5
Weight gain per fattening phase/pig (kg)		
Starter phase (18 days)	9.8 ± 1.9	9.4 ± 1.6
Grower phase (45 days)	30.4 ± 3.5	29.9 ± 4.3
Finisher phase (62 days)	51.1 ± 7.1	50.7 ± 11.7
Entire fattening (125 days)	91.2 ± 6.9	90.1 ± 11.3
Daily weight gain (g)		
Starter phase (18 days)	544 ± 108	525 ± 88
Grower phase (45 days)	675 ± 78	664 ± 95
Finisher phase (62 days)	824 ± 115	818 ± 189
Entire fattening (125 days)	730 ± 55	721 ± 91

**Table 4:** Summary of daily feed intake and feed conversion during the three phases of pig fattening.

Variable	cDDGS (*n* = 6)	Control (*n* = 6)
Net feed intake during fattening phases (kg)		
Starter phase (18 days)	598	606
Grower phase (45 days)	2380	2432
Finisher phase (62 days)	4855	5094
Entire fattening period (125 days)	7833	8132
Feed intake per pig per fattening phase (kg)		
Starter phase (18 days)	18.7 ± 1.1	18.9 ± 1.0
Grower phase (45 days)	74.4 ± 1.0 a	76.0 ± 1.6 b
Finisher phase (62 days)	156.6 ± 5.3 A	164.3 ± 5.4 B
Entire fattening period (125 days)	244.8 ± 7.0 A	254.1 ± 7.6 B
Feed to gain ratio (kg/kg)		
Starter phase (25 days)	1.97 ± 0.33	2.06 ± 0.37
Grower phase (35 days)	2.45 ± 0.31	2.54 ± 0.35
Finisher phase (50 days)	3.07 ± 0.42	3.24 ± 1.52
Entire fattening period (110 days)	2.77 ± 0.18 a	2.91 ± 0.41 b

Within rows, means denoted by different letters are significantly different at ab *p* < 0.05, AB *p* < 0.01.

**Table 5:** Summary of the slaughter values, SEUROP system carcass classifications, and mean prices of pig carcasses (means ± SDs).

Variable	cDDGS (*n* = 31)	Control (*n* = 31)
Live weight (kg)	106.4 ± 7.9	105.5 ± 12.5
Carcass weight (kg)	81.9 ± 6.1	80.3 ± 9.5
Slaughter value (%)	77.0 ± 0.9 a	76.1 ± 2.5 b
Back fat thickness (mm)	16.6 ± 4.6	15.4 ± 4.8
Loin depth (mm)	61.8 ± 5.9 a	56.5 ± 10.9 b
Meatiness (%)	56.4 ± 2.7	55.8 ± 3.3
% of carcasses in S class	6.45	3.23
% of carcasses in E class	70.97	54.84
% of carcasses in U class	19.35	25.81
% of carcasses in R class	3.23	12.90
% of carcasses in P class	0.00	3.23
Carcass price/1 kg (PLN/USD)	5.43 ± 0.16/1.47 ± 0.04 a	5.29 ± 0.38/1.43 ± 0.10 b

ab Within rows, means denoted by different letters are significantly different at *p* < 0.05.

**Table 6:** The cost of nutrition and profitability of pig fattening.

Variable	cDDGS	Control	Percentage (%) Increase/Decrease *
Starter mix price (PLN/USD) **	1105.00/299.46	1133.00/307.05	−2.47%
Starter phase cost	660.79/179.08	686.60/186.07	−3.76%
Starter phase cost per pig	20.65 ± 1.18/5.60 ± 0.32	21.46 ± 1.10/5.81 ± 0.30	−3.76%
Starter phase cost of 1 kg of weight gain	2.11 ± 0.16/0.57 ± 0.10	2.27 ± 0.41/0.62 ± 0.11	−7.08%
Grower mix price (PLN/USD) **	872.00/236.31	898.00/243.36	−2.90%
Grower phase cost	2075.36/562.43	2183.94/591.85	−4.97%
Grower phase cost per pig	64.86 ± 0.84/17.58 ± 0.23 A	68.25 ± 1.41/18.50 ± 0.38 B	−4.97%
Grower phase cost of 1 kg of weight gain	2.14 ± 0.27/0.58 ± 0.07	2.28 ± 0.30/0.62 ± 0.08	−6.43%
Finisher mix price (PLN/USD) **	765.00/207.32	792.00/214.63	−3.41%
Finisher phase cost	3714.08/1006.52	4034.45/1093.35	−7.94%
Finisher phase cost per pig	116.06 ± 4.05/31.45 ± 1.1 A	126.08 ± 4.24/34.17 ± 1.14 B	−7.94%
Finisher phase cost of 1 kg of weight gain	2.35 ± 0.32/0.64 ± 0.09	2.57 ± 1.20/0.70 ± 0.33	−8.59%
Total cost of fattening (PLN/USD) **	6450.23/1748.03	6904.98/1871.27	−6.59%
Total cost of fattening per pig	201.57 ± 5.789/54.63 ± 1.57 A	215.78 ± 6.45/58.48 ± 1.75 B	−6.59%
Total cost of 1 kg of weight gain	2.28 ± 0.15/0.62 ± 0.04 A	2.47 ± 0.35/0.67 ± 0.09 B	−7.71%
Purchase price of piglets/pig (PLN/USD)	150.75/40.85	152.88/41.43	−1.39%
Grand total (expenditures)/pig (PLN/USD)	352.32/95.48	368.66/99.91	−4.43%
Total carcass price (PLN/USD) **	444.14/120.36	424.83/115.13	−4.54%
Simplified direct surplus/pig (PLN/USD)	91.82/24.88	56.17/15.22	+63.47%

* Percentage increase/decrease shows the percentage increase or decrease in the treatment group relative to the control group. Within rows, means denoted by different letters are significantly different at ab *p* < 0.05; AB *p* < 0.01. ** Currency exchange rate (National Bank of Poland): USD 1 = PLN 3.69, as of 10 February 2021.

**Table 7:** Main physicochemical parameters (means ± SDs) of raw *longissimus dorsi* muscle and back fat quality.

Variable	cDDGS (*n* = 15)	Control (*n* = 15)
*Longissimus dorsi* muscle		
pH24h	5.65 ± 0.03	5.67 ± 0.04
Lightness (L*)	54.21 ± 0.57 A	56.77 ± 1.82 B
Color channel a*	3.95 ± 0.63	3.79 ± 0.71
Color channel b*	12.09 ± 0.54	12.34 ± 0.59
Cutting force (N)	34.54 ± 5.97	33.15 ± 7.39
Work of shear (N/s)	177.55 ± 37.03	166.35 ± 36.81
Hardness (N)	51.31 ± 15.50 A	35.56 ± 11.12 B
Adhesiveness (g/s)	−16.47 ± 13.21	−17.30 ± 13.48
Springiness	0.28 ± 0.02	0.29 ± 0.04
Cohesiveness	0.39 ± 0.03	0.40 ± 0.04
Chewiness (N)	5.59 ± 1.792 a	4.19 ± 1.69 b
Resilience	0.21 ± 0.03 A	0.18 ± 0.02 B
Free water content (%)	1.20 ± 0.18 a	1.00 ± 0.08 b
Dry matter (%)	27.30 ± 0.08 A	27.07 ± 0.04 B
Protein (%)	23.72 ± 0.12 a	23.06 ± 0.46 b
Crude fat (%)	1.25 ± 0.06 a	1.09 ± 0.07 b
Back fat		
Lightness (L*)	76.98 ± 1.44	76.57 ± 1.46
Color channel a*	−0.64 ± 0.69	−0.55 ± 1.02
Color channel b*	10.24 ± 1.40	10.32 ± 1.00
Hardness (g)	248.44 ± 65.30	254.45 ± 75.39
Adhesiveness (g/s)	−36.84 ± 18.37	−36.71 ± 17.68
Springiness	0.31 ± 0.07	0.39 ± 0.32
Cohesiveness	0.31 ± 0.10	0.28 ± 0.11
Chewiness (N)	27.05 ± 16.49	30.31 ± 28.13
Resilience	0.21 ± 0.08	0.19 ± 0.07

Within rows, means denoted by different letters are significantly different at ab *p* < 0.05; AB *p* < 0.01.

**Table 8:** Main physicochemical characteristics of the roasted *longissimus dorsi* muscle (means ± SDs).

Variable	cDDGS (*n* = 15)	Control (*n* = 15)
Lightness (L*)	68.83 ± 2.86	70.33 ± 1.60
Color channel a*	0.71 ± 0.81	0.69 ± 0.65
Color channel b*	11.90 ± 0.52 A	13.33 ± 0.90 B
Thermal drip (%)	30.80 ± 1.72 A	25.8 ± 2.91 B
Cutting force (N)	67.58 ± 9.48	61.26 ± 14.23
Work of shear (N/s)	305.56 ± 49.67 a	263.73 ± 75.15 b
Hardness (N)	163.97 ± 24.00	154.01 ± 13.46
Adhesiveness (g/s)	−2.34 ± 1.12	−2.81 ± 3.32
Springiness	0.50 ± 0.05	0.48 ± 0.06
Cohesiveness	0.46 ± 0.03 a	0.49 ± 0.04 b
Chewiness (N)	39.78 ± 10.23	37.36 ± 6.00
Resilience	0.15 ± 0.02 a	0.16 ± 0.02 b
Free water content (%)	0.83 ± 0.09	0.82 ± 0.12
Dry matter (%)	38.36 ± 0.07 A	37.15 ± 0.32 B
Protein (%)	34.25 ± 0.48 A	31.43 ± 0.13 B
Crude fat (%)	2.21 ± 0.08 a	2.42 ± 0.08 b

Within rows, means denoted by different letters are significantly different at ab *p* < 0.05; AB *p* < 0.01.

**Table 9:** Main sensory parameters (means ± SDs) of roasted *longissimus dorsi* muscle analyzed by the seven-person panel.

Variable	cDDGS (*n* = 7)	Control (*n* = 7)
General appearance	4.86 ± 0.24	4.79 ± 0.39
Color	3.64 ± 0.38	3.50 ± 0.50
Structure	3.64 ± 0.48	3.71 ± 0.76
Smell (intensity)	3.79 ± 0.27	3.93 ± 0.84
Smell (desirability)	4.00 ± 0.71	4.07 ± 0.93
Juiciness	3.86 ± 0.38	3.71 ± 0.76
Fragility	3.79 ± 0.39	3.93 ± 0.61
Taste (intensity)	3.64 ± 0.38	3.64 ± 0.48
Taste (desirability)	3.86 ± 0.24	3.79 ± 0.70
General assessment	3.93 ± 0.12	3.89 ± 0.31

The scale of assessment from 1 to 5 (1 = lowest score).

## Data Availability

Original data available upon reasonable request.
